# Supplementary figures and images for: Identification of HMMR as a prognostic biomarker for patients with lung adenocarcinoma via integrated bioinformatics analysis
Source: PeerJ. 2021 Dec 22;9:e12624. doi: 10.7717/peerj.12624 (PMC8710063; doi:10.7717/peerj.12624)

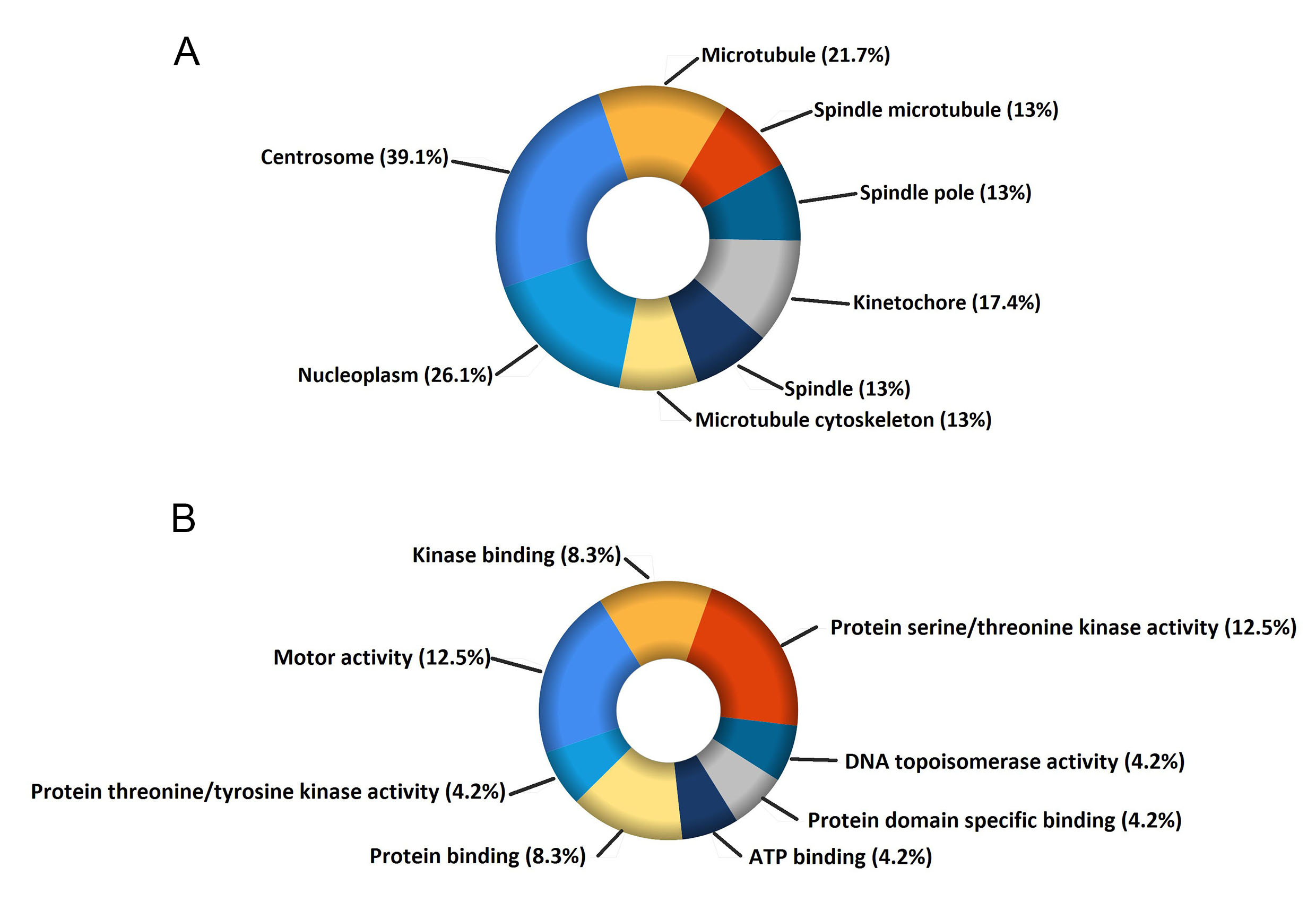

Supplement: Supplemental Information 10 — (A) Cellular component (A) and molecular function enrichment analyses (B) were performed for the 24 DEGs in key module. [file peerj-09-12624-s010.png]

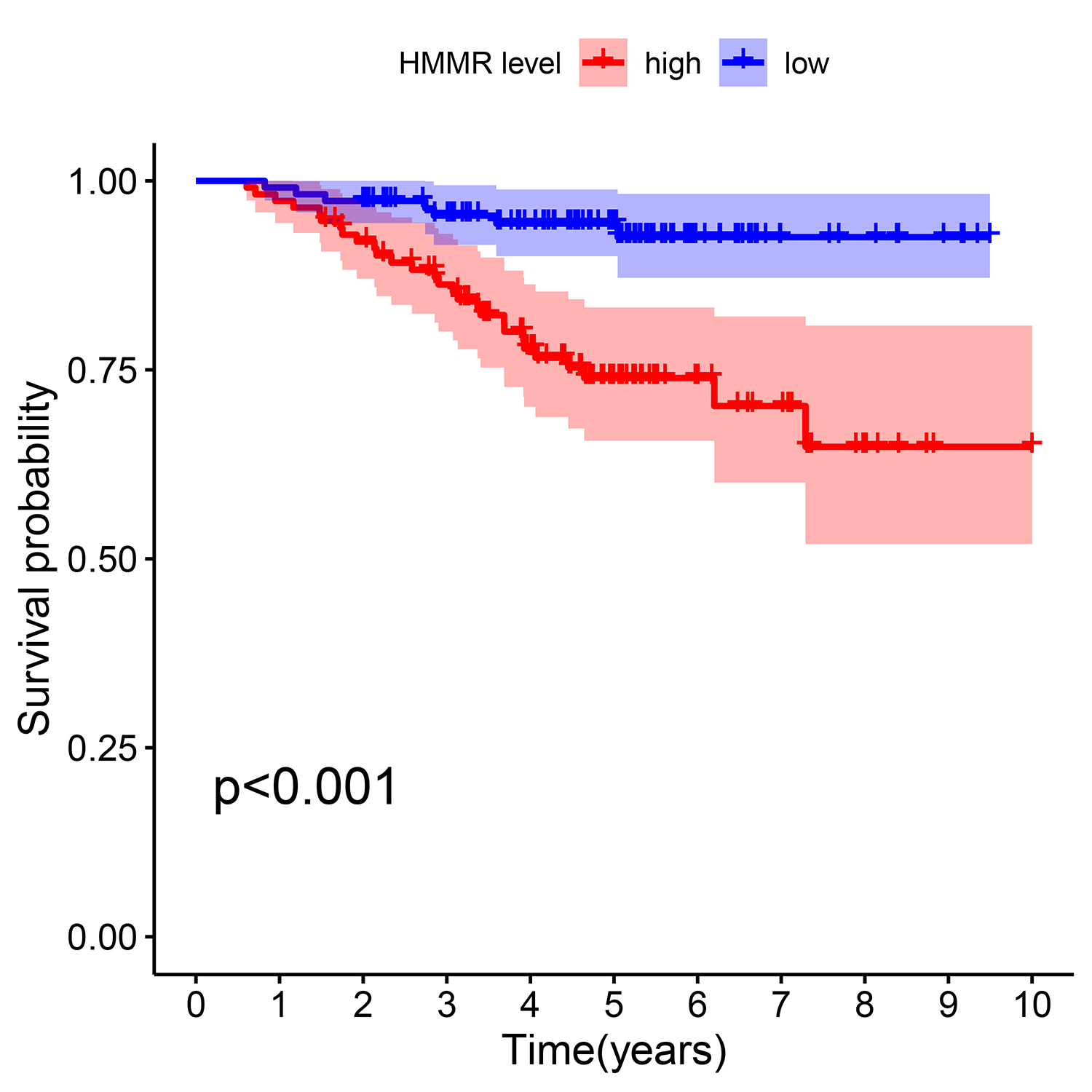

Supplement: Supplemental Information 11 — High HMMR expression was significantly indicated worse overall survival in LUAD patients. [file peerj-09-12624-s011.png]
